# Natural Compounds as Therapeutic Agents: The Case of Human Topoisomerase IB

**DOI:** 10.3390/ijms22084138

**Published:** 2021-04-16

**Authors:** Alessio Ottaviani, Federico Iacovelli, Paola Fiorani, Alessandro Desideri

**Affiliations:** 1 Department of Biology, University of Rome Tor Vergata, Via Della Ricerca Scientifica, 00133 Rome, Italy; federico.iacovelli@uniroma2.it (F.I.); paola.fiorani@uniroma2.it (P.F.); desideri@uniroma2.it (A.D.); 2 Institute of Translational Pharmacology, National Research Council, CNR, Via Del Fosso del Cavaliere 100, 00133 Rome, Italy

**Keywords:** topoisomerase, cancer, natural products

## Abstract

Natural products are widely used as source for drugs development. An interesting example is represented by natural drugs developed against human topoisomerase IB, a ubiquitous enzyme involved in many cellular processes where several topological problems occur due the formation of supercoiled DNA. Human topoisomerase IB, involved in the solution of such problems relaxing the DNA cleaving and religating a single DNA strand, represents an important target in anticancer therapy. Several natural compounds inhibiting or poisoning this enzyme are under investigation as possible new drugs. This review summarizes the natural products that target human topoisomerase IB that may be used as the lead compounds to develop new anticancer drugs. Moreover, the natural compounds and their derivatives that are in clinical trial are also commented on.

## 1. Introduction

Humankind has faced numerous challenges for its survival, even when the challenge was an invisible enemy such as viruses, bacteria and other pathogens. Nature has always provided help in order to allow its survival, and natural products (NPs) have been one solution of human health problems [1,2]. Even nowadays, despite the advent of the pharmaceutical industry, the availability of synthetic compound libraries and the power of the high-throughput screening, scientists find it useful to look at nature as a source of drugs [3,4]. 

The understanding of the power of NPs as a source of drugs started in the 19th century with the isolation of morphine from *Papaver somniferum*, used as an analgesic and sleep-inducing agent that, today, has developed into codeine, a painkiller [5,6]. The most famous drug of natural origin is probably salicylic acid, initially called salicin, extracted from the bark of the willow tree *Salix alba* [4,7]. Salicylic acid was the first NP produced in a large scale by chemical synthesis in 1853 and gave rise to the famous drug aspirin [8]. Additional examples are anti-malaria compounds such as artemisinin from the Chinese herb *Artemisia annua*, used to treat the malaria-causing parasites *Plasmodium falciparum*, and quinine, used since 2004 when it was approved by the US Food and Drug Administration (FDA), isolated from the bark of *Cinchona succiruba* [4,7]. 

Several NPs have been found to display antitumor activity [9]. From bacterial sources, we can list daunorubicin, an anthracycline from *Streptomyces peucetius* [10,11], and its semi-synthetic derivate doxorubicin, which acts intercalating in DNA and blocking human topoisomerase II [12,13]. From plants, we can find vincristine and vinblastine, two terpenes extracted from *Catharanthus roseus* [14,15], that inhibit the mitosis, binding to microtubules [16,17,18]. Another important antitumor agent is camptothecin (CPT), extracted from the bark of the Chinese tree *Camptotheca acuminata*, and the soluble derivatives irinotecan and topotecan, both efficient topoisomerase I poisons [19,20]. 

**Figure 1:**
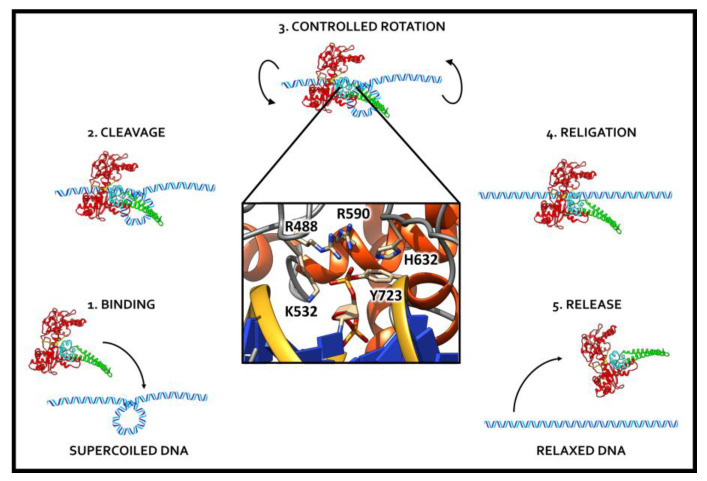
Schematic representation of the catalytic cycle of hTopI. Once the enzyme binds a supercoiled DNA (1), the cleavage step occurs (2), followed by the controlled rotation of the cleaved strand (3) and by a religation event (4) and the release of the unwound substrate (5).

DNA topoisomerases are a class of ubiquitous enzymes identified for the first time in 1971 in *Escherichia coli* by James C. Wang [21]. Subsequently, this enzyme was found in nuclear extracts from eukaryotic mouse embryo cells by Champoux and Dulbecco [22]. The enzyme is able to relax supercoiled DNA to introduce negative or positive supercoils into DNA and to decatenate circular DNA. Indeed, DNA topoisomerases deal with all the cellular processes that involve DNA topological issues and, in human cells, are involved in regulating several fundamental processes: DNA replication, transcription and chromosome segregation [23]. Human topoisomerases (hTops) are grouped into class I (hTopI) and II (hTopII), according to their ability to cut one or both DNA strands to release the constrains and unwind supercoiled DNA [24]. In the hTopI enzyme, catalysis occurs through a tyrosine residue, located in the catalytic pocket at the C-terminal, which undergoes a nucleophilic attack on the phosphodiester bond of DNA, forming a transient phosphotyrosyl bond with the 3’ or 5’ DNA break (Figure 1). These two different types of bonds define two subclasses of the enzyme named A and B when they bind the 3’ or 5’, respectively. Once the rotation has been completed, the religation step can occur, bringing the reconstitution of the phosphodiester backbone and the consequent release of the enzyme from the DNA (Figure 1) [25,26,27,28]. It is worth noting that DNA unwinding is driven by torsional strain, rather than powered by ATP hydrolysis [28,29]. 

## 2. Human DNA Topoisomerase IB as the Tumor Target

**Figure 2:**
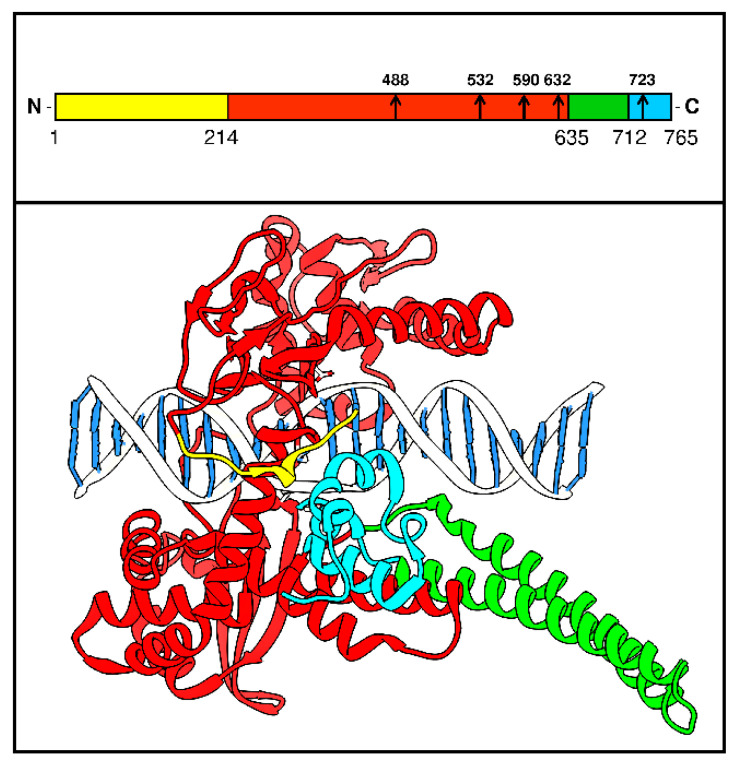
Structure of hTopIB. Top panel schematic representation of the hTopIB domains. The N-terminal domain in yellow (1–214), the core in red (215–635), the linker in green (636–712) and the C-terminal domain in light blue (713–765). The arrows represent the amino acids forming the active site. Bottom panel is the 3D structure of the enzyme, where the domains are represented in the same color.

HTopIB is a 91-KDa protein, made up of 765 amino acids, divided into 4 domains: The N-terminal, the core, the linker and the C-terminal domain (Figure 2 Top). The N-terminal domain (1–214) allows the enzyme nuclear localization [30] and is involved in the modulation of the noncovalent enzyme–DNA interactions [31]. The core domain (215–635) is highly conserved and is directly involved in the binding of the DNA substrate [32,33]. Single mutations in this domain, such as glutamine 418, induce a different DNA-binding specificity and modulate the enzyme–drug interactions [34]. The linker domain (636–712) has a fundamental role in the catalytic mechanism controlling the rotation of the free DNA strand around the cleavage site [35,36]. Indeed, mutations that alter the flexibility of the linker perturb the enzyme sensitivity to the drugs targeting the enzyme [37,38,39,40]. The C-terminal domain (713–765) contains Tyr 723, which undergoes the nucleophilic attack to the substrate and forms together with Arg 488, Lys 532, Arg 590 and His 632 the catalytic site [23,41,42]. The mutation of Gly 717, located in this domain, causes a slight rearrangement of the active site and perturbs the drug binding site [43].

There are two different types of drugs that can affect hTopIB catalysis: poisons and inhibitors [44,45,46]. The poisons are compounds that lead to the stabilization of a ternary complex between the enzyme, DNA and drug itself, turning the enzyme into a poison. In detail, the catalytic cycle consists in the cutting of a single DNA strand, strand rotation and, finally, religation of the relaxed substrate. In the presence of a poisoning drug that intercalates DNA in correspondence to the cleavage site, the enzyme is inhibited to undergo the religation step. The persistence of hTopIB on the nicked DNA leads to the stalling and collapse of the replication fork and to the formation of DNA double-stranded breaks on the enzyme cleavage site activating apoptosis and inducing cell death [47]. The inhibitors work in a simpler manner; they inhibit the cleavage of the DNA by the enzyme or prevent the binding to DNA. In this case, the persistence of supercoiled regions during cell replication lead to the stall of the replication fork, the formation of DNA single-stranded breaks and a consequent genomic damage that brings the cell to its death.

Poisons have clinical relevance, and their efficient cytotoxic effect is demonstrated by the use of CPT, the first discovered hTopIB poison [19,48,49]. CPT is an E-ring lactone that reversibly interacts with both DNA and hTopIB, intercalating between the DNA bases after the DNA cleavage has occurred, trapping the enzyme on the DNA and bringing the cells to death. The CPT derivatives, irinotecan and topotecan, are in clinical use, but they have several side effects [44]. 

The arising importance of hTopIB as a tumor target has pushed researchers to look for novel natural sources to be used as lead compounds to selectively poison hTopIB without the side effects observed for the CPT derivatives [20]. In a previous manuscript, we reviewed the natural compounds targeting hTops up to 2012 [50]; here, we review the natural compounds reported to target hTopIB from 2012 to now and the modified natural compounds that are in clinical trials.

## 3. Natural Compounds with In Vitro and In Vivo Activity on hTopIB

NPs with in vitro and in vivo antitumor activity targeting hTopIB are listed in Table 1.

### 3.1. Epigallocatechin-3-Gallate

**Figure 3:**
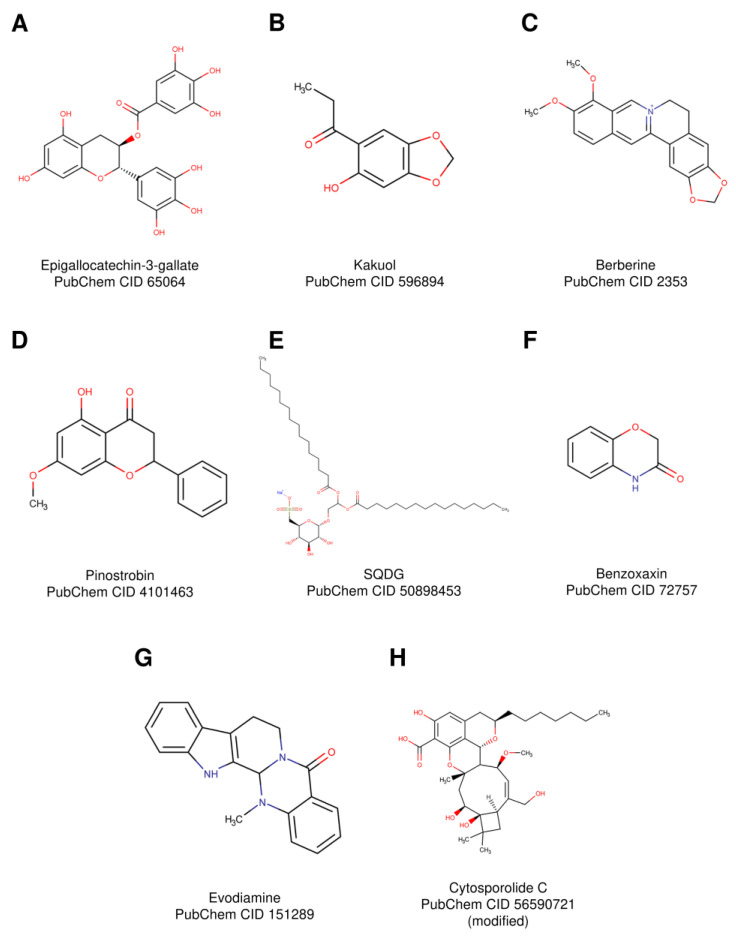
Structures of natural products with in vivo and in vitro activity against hTopIB. (**A**) Epigallotechin-3-gallate, (**B**) Kakuol, (**C**) Berberine, (**D**) Pinostrobin, (**E**) Sulfonoquinovosyl diacylglyceride SQDG, (**F**) Benzoxaxin, (**G**) Evodiamine and (**H**) Cytosporolide C. Structures are represented by Marvinsketch, as reported on PubChem.

Epigallocatechin-3-gallate (EGCG) (Figure 3A) is a major polyphenolic constituent of green tea extracted from *Camellia sinensis* leaves [59]. Over the years, this plant has received a lot of attention for the health benefits associated with green tea consumption, such as antioxidant effects, cancer chemoprevention, cardiovascular health improvement, weight loss enhancement and skin protection from the damage caused by ionizing radiation. Nowadays, several laboratories have demonstrated that EGCG possess cancer therapeutic effects, and Mukhtar’s group at the University Hospitals of Cleveland has shown that this compound has a dose-dependent inhibitory effect on several human carcinoma cell lines [60]. In normal cell lines, EGCG does not have any cytotoxic effect, as tested by a viability assay [60]. EGCG has a complex mechanism of action, and it has several targets, such as nuclear factor kappa light-chain enhancer of activated B cells (NF-kB) [61], vascular endothelial growth factor (VEGF) [62] and hTopIB [51]. A significant inhibition of hTopoIB activity but not hTopII was observed through a relaxation assay [51].

### 3.2. Kakuol 

Kakuol (Figure 3B) is a metabolic oxidation product isolated from the rhizomes of *Asarum sieboldii*. Extracts from this plant, have antalgic, anti-inflammatory, anticonvulsive, antitussive, antiallergic and antitumoral activities [63]. Studies of interactions of this compound and its derivatives with hTopIB demonstrate that kakuol is a catalytic inhibitor of this enzyme. The compound inhibits a cleavage reaction, and the effect is enhanced preincubating the drug with the enzyme. The effect is due to the inhibition of the catalytic activity and not to the prevention of DNA binding, as shown by the EMSA assay [64].

### 3.3. Berberine 

*Coptis chinensis* and *Berberis vulgaris* are two Chinese plants that produce a natural quaternary alkaloid called berberine [65]. This compound (Figure 3C) can be found in roots, rhizome and the stem bark of plants, and it has been used since ancient times in Chinese medicine. Berberine has several positive effects, such as antimicrobial [66], anti-inflammatory [67], anti-arhythmic [68] and antitumor activity [52]. Regarding antitumor activity, it has been observed that berberine and its derivatives are able to inhibit hTopIB in a dose-dependent manner. The inhibition is more evident when hTopIB is preincubated with the compound [69]. The drug works as a catalytic inhibitor, since the enzyme is able to bind, but not to cleave, the DNA in the presence of berberine.

### 3.4. Pinostrobin

Pinostrobin (Figure 3D) is a flavonoid found in honey and in some dietary plants and is used as a natural food supplement. The compound has shown antimicrobial [70], anti-inflammatory [71], antioxidant [72] and antiproliferative properties [73]. Pinostrobin has been studied by Jadaun et al., who suggested that the compound forms a ternary complex with hTopIB and DNA [53]. The in vitro catalytic assay and in silico analysis indicate that the binding of pinostrobin occurs at the interface of hTopIB and DNA in a CPT-like manner. The authors propose that the compound can be used as the lead compound to develop new hTopIB poisons.

### 3.5. Sulfonoquinovosyl Diacylglyceride

Sulfonoquinovosyl diacylglyceride (SQDG), identified for the first time by Benson and coworkers in photosynthetic bacteria and higher plants [74], is a plant sulfolipid isolated from *Azadirachta indica*, showing antibacterial, antiviral [75] and antileukemic activity [76]. SQGD (Figure 3E) is able to inhibit the hTopIB enzyme, as evaluated by the relaxation assay and by a cleavage assay on a radiolabeled oligonucleotide [76]. The results indicated that the compound acts as a catalytic inhibitor. The in vitro test on acute lymphoblastic/lymphocytic leukemia cell lines overexpressing hTopIB and in vivo experiments in nude mice demonstrate that SQDG treatment delays tumor growth and reduces the expression of cell proliferation markers.

### 3.6. Benzoxazines

Benzoxazines, such as 1,4-benzoxazin-3-ones and 2,4-Dihydroxy-1,4-benzoxazin-3-one (Figure 3F), present in maize [77], wheat and rye [54] are a group of molecules showing antimicrobial and antitumor activity [78,79]. In a recent work, Foto et al. demonstrated that benzoxazines and their derivatives act as hTopIB inhibitors, interfering with the binding of the enzyme to the DNA [55]. According to relaxation assay and EMSA experiments, the DNA-binding capacity of hTopoIB is reduced by benzoxazines in a dose-dependent concentration, suggesting a possible use of these molecules as a lead compound to develop new drugs for cancer treatment.

### 3.7. Evodiamine

The *Evodia rutaecarpa* fruit, officially listed in the Chinese Pharmacopoeia, has been used as an analgesic, anti-inflammatory and in the treatment of hypertension, suggesting a beneficial use for a variety of therapeutic applications [80,81]. Researchers have isolated from this fruit an alkaloid named evodiamine (EVO) reported in Figure 3G, that has shown in vitro anticancer properties [82]. EVO inhibits hTopIB, as shown by a relaxation assay on the supercoiled DNA [83]. The proposed mechanism of action is an inhibition of the enzyme in a CPT-like manner. Chan et al. [56] reported that this compound is able to trap hTopIB on DNA to form a ternary covalent complex. 3H-thymidine-labeled cells were treated with EVO, and the cell extracts were subjected to KCl/SDS that induces protein but not DNA precipitation, except when it is linked to a protein. The amount of precipitated DNA, evaluated by autoradiography, is proportional to the amount of the hTopIB-EVO-DNA complex, indicating a DNA EVO-trapping activity. The authors suggest that the compound acts by stabilizing the covalent complex between hTopIB and DNA, forming a barrier to the DNA replication fork and converting the ternary covalent complex into a cell poison.

### 3.8. Cytosporolide C

Cytosporolide C (Cyto-C) (Figure 3H) is a NP isolated from the fungus *Cytospora* spp. [84]. This compound has antimicrobial activity [85], but a novel bioactivity as antiproliferative compound has been recently demonstrated, suggesting a potential use as an anticancer drug [86]. The results demonstrate that Cyto-C inhibits the hTopIB relaxation of a supercoiled DNA substrate and has an antiproliferative activity against A549 (non-small-cell lung cancer cells), HCT-116 (human colon cancer cells) and MCF-7 (breast cancer cells) cell lines. Cyto-C is an interesting hTopIB-specific inhibitor and a promising lead compound for the development of new drugs for cancer treatment.

## 4. HTopIB Inhibition by Natural Compounds Coordinated with Metals

**Figure 4:**
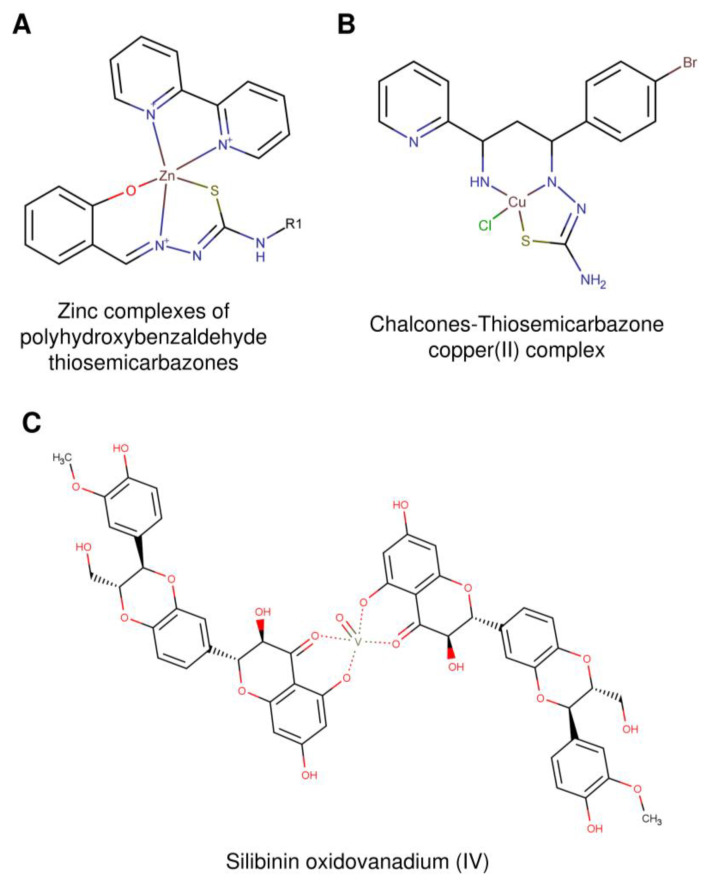
Structures of natural compounds coordinated with metals with in vitro activity against hTopIB. (**A**) Polyhydroxybenzaldehyde thiosemicarbazones zinc complex, (**B**) chalcone thiosemicarbazones copper(II) complex and (**C**) silibinin oxidovanadium(IV) complex. All structures are represented by Marvinsketch.

Some NPs display positive properties only upon metal coordination [87], and among them, there are some hTopIB inhibitors, reported in Figure 4 and Table 2.

Thiosemicarbazones are fundamental compounds for regulating plants growth [57]. Zinc complexes of polyhydroxybenzaldehyde thiosemicarbazones (Figure 4A) interact with hTopIB [91]. Incubation of the metal complex with hTopIB and DNA gives rise to two different modes of actions: one concerns the binding of the metal complex to DNA, while the other one involves the binding to the enzyme. When the drug is incubated with hTopIB before adding the supercoiled DNA substrate, there is an inhibition of the enzyme activity stronger than preincubating the compound with DNA. These experiments are an indication that the binding of the metal complex to hTopIB is the main inhibition mechanism. 

Chalcones are intermediate products in flavonoids synthesis [92], found mainly in *Piper methysticum* [58], *Boesen-bergia rotunda* [93] and *Lophira alata* [94]. Chalcones-derived thiosemicarbazones (Figure 4B) are efficient in inhibiting hTopoIB only when coordinated to a copper atom [88]. This complex, when preincubated with the enzyme, prevent the binding to DNA, as demonstrated by the EMSA and relaxation assay, indicating that the copper complex acts as an inhibitor and not as a poison. 

A similar behavior is observed with the flavonoid silibinin, extracted from *Silybum marianum* [95,96] and chrysin from *Passiflora caerulea* [97]. The two flavonoids did not show any effect on hTopIB, but silibinin inhibits the enzyme when forming oxidovanadium(IV) complexes [98]. The results demonstrate that the silibinin oxidovanadium(IV) complex (Figure 4C) acts by preventing the formation of the enzyme–DNA complex. The compound has a positive effect on human colon cancer cell line HT-29, as tested by a cell viability assay, suggesting its possible use in antitumor treatments.

## 5. Natural Compounds from Marine Organism 

Due to their different growing environments, marine and, in particular, Antarctic organisms have developed NPs with novel characteristics that deserve to be investigated. Hereafter, the effects of these NPs targeting hTopIB are presented in Table 3.

### 5.1. Bacillosporin C

**Figure 5:**
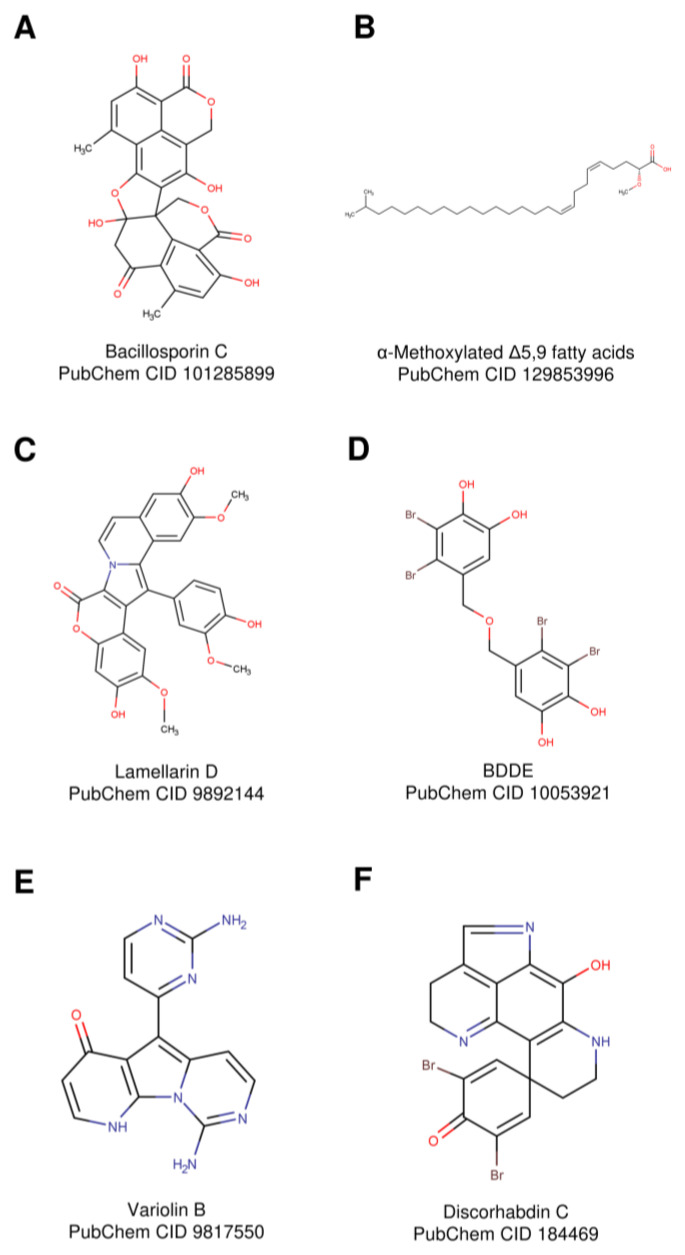
Structures of marine natural products with in vitro and/or in silico activity against hTopIB. (**A**) Bacillosporin C, (**B**) alpha-methoxylated δ5,9 fatty acids, (**C**) Lamellarin D, (**D**) Bis(2,3-dibromo-4,5-dihydroxybenzyl) ether (BDDE), (**E**) Variolin B and (**F**) Discorhabdin C. The structures are represented by Marvinsketch, as reported on PubChem.

Bacillosporin C (Bac-C) (Figure 5A) is an oxaphenalenone, an important class of phenolic natural products, isolated from fungi, such as *Penicillium purpurogenum* [89]. *P. purpurogenum* has the ability to synthesize a variety of substances with antibacterial activity and inhibitory effects on several human cancer cell lines. The strains with large biotechnological potential are mutants resistant to antibiotics [104]. An example is the marine G59 strain producing Bac-C. Bac-C can target hTopIB, as shown by an experimental bioassay and docking simulation [51]. The screening of 128 compounds, by docking them on the hTopI–DNA complex, permitted the selection of compounds found to be hTopIB inhibitors through a relaxation assay on a supercoiled DNA substrate. The researchers did not investigate the mechanism of action; thus, the compounds cannot be classified as catalytic inhibitors or poisons.

### 5.2. Alpha-Methoxylated δ5,9 Fatty Acids

Sponges are the source of new phospholipid fatty acids, having long chains (C23–C30) with no counterpart in the terrestrial world [105]. α-Methoxylated Δ5,9 fatty acids (Figure 5B) were extracted and isolated from the Caribbean sponge *Asteropus niger* [106]. The compound is able to inhibit hTopIB with a mechanism of action different from that displayed by CPT. Indeed, the compound does not bind to the DNA-hTopIB complex but directly interacts with the enzyme, preventing the catalytic tyrosine to do the nucleophilic attack on the DNA phosphate bond [106].

### 5.3. Lamellarin D

Lamellarin D (LAM-D) (Figure 5C) is a hexacyclic marine alkaloid isolated for the first time from a mollusk of the genus *Lamellaria* [90], with a cytotoxic effect on the tumor cells lines [107,108]. On the basis of its chemical structure and on a molecular modeling analysis, it has been suggested that LAM-D can bind to DNA and interact with hTopIB, affecting its catalytic mechanism [109]. LAM-D inhibits the relaxation of supercoiled DNA in a dose-dependent manner. The researchers, investigating the cleavage/religation reaction, demonstrated that LAM-D stabilizes the DNA–enzyme complex, turning the enzyme into a poison, acting like CPT. LAM-D has appeared as a new potent hTopIB poison [99] that should be further investigated to develop a new non-CPT derivatives drug.

### 5.4. Bis(2,3-dibromo-4,5-dihydroxybenzyl) Ether 

Marine bromophenols, found in sponges and algae have been always attracted food and pharmaceutical company due to their multiple bioactivities, such as antioxidant [110], antimicrobials [111] and antidiabetic activity [112]. Among marine bromophenols, bis (2,3-dibromo-4,5-dihydroxybenzyl) ether (BDDE) (Figure 5D), isolated from marine algae *Leathesia nana* and *Rhodomela confervoides*, has been shown to inhibit the proliferation of several tumor cells lines and induce apoptosis in human myelogenous leukemia cell line K562 [113]. A relaxation assay of a supercoiled DNA in the presence of different amounts of BDDE indicates that the compound inhibits hTopIB in a dose-dependent manner. BDDE is not able to trap the enzyme–DNA cleavable complex, and no nicked DNA is observed [113]. These data suggest that BDDE behave as a catalytic inhibitor rather than a poison.

### 5.5. Variolin B

Variolin B (Figure 5E) is a NP from the Antarctic sponge *Kirckpatrickia variolosa* and is reported to have antitumor and antiviral properties [100]. Due to its planar structure and the presence of a central aromatic ring, a cytotoxic effect through DNA intercalation has been proposed [114]. The more soluble derivative deoxyvariolin B has been developed and tested as an hTopIB inhibitor. This compound partially affects hTopIB activity, inhibiting the relaxation of supercoiled DNA [115]. This result suggests the possibility of developing new variolin B derivatives with improved antitumor efficacy.

### 5.6. Discorhabdins

Discorhabdins (Figure 5F) are a subclass of pyrroloiminoquinone alkaloids [116] associated with the chemical defense of the Antarctic sponge *Latrunculia biformis*, turning its color from green to brown to deter predators such as sea stars [101]. These NPs have shown a strong anticancer activity in different cancer types, such as human colon cancer, adenocarcinoma and leukemia, but its mechanism of action is still unknown [117]. Li et al. suggested hTopIB as the possible target applying a structure-based docking approach [118]. This result comes from a computational study but appears promising and suggests that discorhabdins can be experimentally tested against hTopIB.

## 6. HTopIB Poisons Derived from Natural Compounds in Preclinical and Clinical Trial 

The best characterized NP against hTopoIB is CPT [44,49,102]. Two water-soluble derivatives, both approved by the FDA in 1996, are currently used in clinics, irinotecan for colon carcinomas [119,120] and topotecan for ovarian cancers [121]. Topotecan has been subsequently also approved for small cell lung cancer and, in combination with cisplatin, for stage IV-B cervical carcinoma [103,122]. Another interesting CPT derivative is belotecan [123], approved in South Korea in 2003 for the treatment of non-small cell lung cancer [124,125] and ovarian cancer [126]. New drugs, targeting hTopIB, are also under development, because some tumors show resistance to the currently in use CPT derivatives [127,128]. 

Drugs having a completed or ongoing clinical trial are listed in Table 4 [129].

Camptothecin-20(S)-O-propionate hydrate (CZ48) [130], obtained reacting CPT with propionic anhydride, is in Phase 1 clinical trials to evaluate its dose-limiting toxicities profile. The drug has been administered for the treatment of malignant lymphoma of extranodal and/or solid organ sites and solid tumors. 

LMP744, an indenoisoquinoline with improved characteristics over CPT derivatives [131], is in phase 1 for the treatment of solid tumors and lymphomas. Indenoisoquinolines have a chemical stability larger than CPT derivatives, produce stable DNA-hTopIB cleavage complexes and exhibit a sequence preference for the DNA cleavage sites. The drug has activity against CPT-resistant cell lines and produces irreversible DNA–protein crosslinks. LMP744 exhibits antitumor activity with lower toxicity than other agents in preclinical studies. The treatment of patients with LMP744 is expected to reduce the tumor burden at doses that are well-tolerated. Among indenoisoquinoline derivatives, CYB-L10 is not yet in clinical trial, but preclinical studies indicated an interesting cytotoxicity profile and an hTopIB inhibition higher than CPT [132]. CYB-L10 is active in vitro against 60 clinical cancer cell lines and displays an antitumor efficiency in an HCT-116 xenograft nude mice model with no obvious loss of weight of the body of the mice at a 20-mg/kg dose [132].

Lipotecan^®^, which is the trade name of TLC388, is on a phase II trial to evaluate the drug efficacy and safety in subjects with poorly differentiated neuroendocrine carcinomas [133]. The Abramson Cancer Center of the University of Pennsylvanian is testing erinotecan pegol (NKTR-102), a hTopIB inhibitor polymer conjugate, made up of irinotecan conjugated with polyethylene glycol (PEG), administered to subjects with metastatic and recurrent NSCLC, after the failure of second-line therapy. Luye Pharma Group Ltd. is testing in a phase II trial, irinotecan hydrochloride liposome (LY01610), in patients with extensive-stage small cell lung cancer (SCLC) that progressed after first-line antitumor therapy. Irinotecan liposome is formulated with irinotecan into a liposomal dispersion. The liposome is a unilamellar lipid bilayer vesicle that encapsulates an aqueous space containing irinotecan in a gelated or precipitated state as sucrose octasulfate salt. Administration via a liposomal formulation results in prolonged intratumor exposure at levels above the threshold for antitumor activity.

## 7. Conclusions

Analysis of the literature indicates that several natural compounds are targeting hTopIB, a ubiquitous enzyme involved in several fundamental cellular processes [23,134]. The development of these molecules into selective and efficient antitumor drugs still requires several passages. However, besides the CPT derivatives already in clinical use, there are interesting indenoisoquinoline compounds and additional CPT derivatives that are in clinical trials [129,135]. We believe that attention must be paid to additional natural compounds, such as NPs coming from the marine and Antarctic worlds (Table 3 and Figure 5). This type of environment has selected organisms adapted to extreme life conditions, producing NPs with no counterparts in the terrestrial world [14]. These compounds, chemically different from CPT, may have the potential to overcome the diffuse drug resistance caused by intense and long-lasting treatments of CPT derivatives and offer a more personalized patient treatment [127].

## Figures and Tables

**Table 1:** Natural compounds with in vitro and/or in vivo anti-hTopIB activity.

Compound Name	Source	Type of Inhibitor	Reference
EGCG	*Camellia sinensis*	Not studied	[51]
Kakuol	*Asarum sieboldii*	Catalytic Inhibitor	[52]
Berberine	*Coptis chinensis* and *Berberis vulgaris*	Catalytic Inhibitor	[53]
Pinostrobin	Honey and dietary vegetables	Poison	[54]
SQDG	*Azadirachta indica*	Catalytic Inhibitor	[55]
Benzoxazines	*Capparis sikimensis*	Catalytic Inhibitor	[56]
Evodiamine	*Evodia rutaecarpa*	Poison	[57]
Cytosporolide C	*Cytospora sp*	Not studied	[58]

**Table 2:** Natural compounds coordinated with metals showing an anti-hTopIB activity.

Compound Name	Source	Type of Inhibitor	Reference
Zinc complexes of polyhydroxybenzaldehyde thiosemicarbazones	Plants natural product	Catalytic Inhibitor	[88]
Chalcones-Thiosemicarbazone copper(II) complex	*Piper methysticum*, *Boesen-bergia rotunda*, *Lophira alata*	Catalytic Inhibitor	[89]
Silibinin oxidovanadium (IV)	*Silybum marianum*	Catalytic Inhibitor	[90]

**Table 3:** Natural products of marine origins inhibiting hTopIB.

Compound Name	Source	Type of Inhibitor	Reference
Bacillosporin C	*Penicillium purpurogenum species*	Not studied	[67]
α-Methoxylated Δ5,9 fatty acids	*Asteropus niger*	Catalytic Inhibitor	[99]
Lamellarin D	*Lamellaria* spp.	Poison	[100]
BDDE	*Leathesia nana*, *Rhodomela confervoides*	Catalytic Inhibitor	[101]
Deoxyvariolin B	*Kirckpatrickia variolosa*	Not studied	[102]
Discorhabdins	*Latrunculia biformis*	Not studied	[103]

**Table 4:** Drugs targeting hTopIB under clinical trial [129].

Study	Study Purpose	Time Frame	Sample Size	NCT
Phase I Study Clinical Trial of Camptothecin-20-O-Propionate Hydrate (CZ48) Malignant Lymphoma of Extranodal and/or Solid Organ Site and Solid Tumor	Describe the dose limiting toxicities and adverse event profile of Camptothecin-20-O-Propionate hydrate (CZ48) administered orally every day for 4 weeks	July 2008–February 2020	65 participants	NCT02575638
A Phase I Study Indenoisoquinoline LMP744 in Adults With Relapsed Solid Tumors and Lymphomas	Establish the safety, tolerability and the maximum tolerated dose (MTD) of LMP744 administered intravenously (IV) in patients with refractory solid tumors and lymphomas	February 2017–ongoing (estimated completion October 2022)	53 participants	NCT03030417
Phase II Study Evaluate the Efficacy and Safety of TLC388 (Lipotecan^®^) as Second-line Treatment in Subjects With Poorly Differentiated Neuroendocrine Carcinomas	Evaluate the efficacy and safety of Lipotecan^®^ monotherapy in subjects with poorly differentiated neuroendocrine carcinomas. Only those subjects who have failed to first line chemotherapy	July 2015–ongoing (last update 3 April 2019	23 participants	NCT02457273
Phase II Study Study of Etirinotecan Pegol (NKTR-102) in the treatment of patients with metastatic and Recurrent Non-Small Cell Lung Cancer (NSCLC) after failure of 2nd line treatment.	Estimate the objective response rate (Complete Response or Partial Response, as measured by RECIST version 1.1) for patients with metastatic or recurrent NSCLC being treated with etirinotecan pegol after failure of second-line therapy.	January 2013–ongoing (last update April 2020)	40 participants	NCT01773109
A Phase II Study LY01610 (Irinotecan Hydrochloride Liposome Injection) in Patients with Small Cell Lung Cancer	Evaluate the efficacy and safety of LY01610 in subjects with extensive small cell lung cancer that progressed after first-line anti-tumor therapy	November 2019–Ongoing (estimated completion September 2022)	90 participants	NCT04381910

## References

[1] Newman D.J., Cragg G.M. (2012). Natural products as sources of new drugs over the 30 years from 1981 to 2010. J. Nat. Prod..

[2] Kinghorn A.D., Pan L., Fletcher J.N., Chai H. (2011). The relevance of higher plants in lead compound discovery programs. J. Nat. Prod..

[3] Baker D.D., Chu M., Oza U., Rajgarhia V. (2007). The value of natural products to future pharmaceutical discovery. Nat. Prod. Rep..

[4] Dias D.A., Urban S., Roessner U. (2012). A Historical Overview of Natural Products in Drug Discovery. Metabolites.

[5] Thongphasuk P., Stremmel W., Chamulitrat W. (2009). 2,3-Dehydrosilybin Is a Better DNA Topoisomerase I Inhibitor than Its Parental Silybin. Chemotherapy.

[6] Bhandari M., Bhandari A., Bhandari A. (2011). Recent updates on codeine. Pharm. Methods.

[7] Tarver T. (2014). The Review of Natural Products. J. Consum. Health Internet.

[8] Limmroth V., Katsarava Z., Diener H.-C. (1999). Acetylsalicylic acid in the treatment of headache. Cephalalgia.

[9] Katz L., Baltz R.H. (2016). Natural product discovery: Past, present, and future. J. Ind. Microbiol. Biotechnol..

[10] Giddings L.A., Newman D.J. (2013). Microbial natural products: Molecular blueprints for antitumor drugs. J. Ind. Microbiol. Biotechnol..

[11] Di Marco A., Cassinelli G., Arcamone F. (1981). The discovery of daunorubicin. Cancer Treat. Rep..

[12] Pommier Y., Schwartz R.E., Kohn K.W., Zwelling L.A. (1985). Effects of DNA Intercalating Agents on Topoisomerase II Induced DNA Strand Cleavage in Isolated Mammalian Cell Nuclei. Biochemistry.

[13] Tewey K.M., Rowe T.C., Yang L., Halligan B.D., Liu L.F. (1984). Adriamycin-induced DNA damage mediated by mammalian DNA topoisomerase II. Science.

[14] Cragg G.M., Newman D.J. (2013). Natural products: A continuing source of novel drug leads. Biochim. Biophys. Acta Gen. Subj..

[15] Atanasov A.G., Waltenberger B., Pferschy-Wenzig E.M., Linder T., Wawrosch C., Uhrin P., Temml V., Wang L., Schwaiger S., Heiss E.H. (2015). Discovery and resupply of pharmacologically active plant-derived natural products: A review. Biotechnol. Adv..

[16] Jordan M.A., Thrower D., Wilson L. (1992). Effects of vinblastine, podophyllotoxin and nocodazole on mitotic spindles. Implications for the role of microtubule dynamics in mitosis. J. Cell Sci..

[17] Jordan M.A., Thrower D., Wilson L. (1991). Mechanism of Inhibition of Cell Proliferation by Vinca Alkaloids. Cancer Res..

[18] Wilson L. (1986). Microtubules as Targets for Drug and Toxic Chemical Action: The Mechanisms of Action of Colchicine and Vinblastine. The Cytoskeleton.

[19] Liu L.F., Desai S.D., Li T.K., Mao Y., Sun M., Sim S.P. (2000). Mechanism of action of camptothecin. Annals of the New York Academy of Sciences.

[20] Wall M.E., Mansukh C. (1995). Wani Camptothecin and Taxol: Discovery to Clinic. Cancer Res..

[21] Wang J.C. (1971). Interaction between DNA and an Escherichia coli protein omega. J. Mol. Biol..

[22] Champoux J.J., Dulbecco R. (1972). An activity from mammalian cells that untwists superhelical DNA-a possible swivel for DNA replication (polyoma-ethidium bromide-mouse-embryo cells-dye binding assay). Proc. Natl. Acad. Sci. USA.

[23] Champoux J.J. (2001). DNA topoisomerases: Structure, function, and mechanism. Annu. Rev. Biochem..

[24] Wang J.C. (1991). DNA topoisomerases: Why so many?. J. Biol. Chem..

[25] Soren B.C., Dasari J.B., Ottaviani A., Lacovelli F., Fiorani P. (2019). Topoisomerase IB: A relaxing enzyme for stressed DNA. Cancer Drug Resist..

[26] Cretaio E., Pattarello L., Fontebasso Y., Banedetti P., Losasso C. (2007). Human DNA topoisomerase IB: Structure and functions. Ital. J. Biochem..

[27] Cheng C., Shuman S. (1998). A catalytic domain of eukaryotic DNA topoisomerase I. J. Biol. Chem..

[28] Stewart L. (1998). A Model for the Mechanism of Human Topoisomerase I. Science.

[29] Leppard J.B., Champoux J.J. (2005). Human DNA topoisomerase I: Relaxation, roles, and damage control. Chromosoma.

[30] Alsner J., Svejstrup J.Q., Kjeldsen E., Sørensen B.S., Westergaard O. (1992). Identification of an N-terminal domain of eukaryotic DNA topoisomerase I dispensable for catalytic activity but essential for in vivo function. J. Biol. Chem..

[31] Lisby M., Olesen J.R., Skouboe C., Krogh B.O., Straub T., Boege F., Velmurugan S., Martensen P.M., Andersen A.H., Jayaram M. (2001). Residues Within the N-terminal Domain of Human Topoisomerase I Play a Direct Role in Relaxation. J. Biol. Chem..

[32] Redinbo M.R., Stewart L., Kuhn P., Champoux J.J., Hol W.G. (1998). Crystal structures of human topoisomerase I in covalent and noncovalent complexes with DNA. Science.

[33] Coletta A., Desideri A. (2013). Role of the protein in the DNA sequence specificity of the cleavage site stabilized by the camptothecin topoisomerase IB inhibitor: A metadynamics study. Nucleic Acids Res..

[34] Fiorani P., Chillemi G., Losasso C., Castelli S., Desideri A. (2006). The different cleavage DNA sequence specificity explains the camptothecin resistance of the human topoisomerase I Glu418Lys mutant. Nucleic Acids Res..

[35] Redinbo M.R., Stewart L., Champoux J.J., Hol W.G. (1999). Structural flexibility in human topoisomerase I revealed in multiple non-isomorphous crystal structures. J. Mol. Biol..

[36] Stewart L., Ireton G.C., Champoux J.J. (1999). A Functional Linker in Human Topoisomerase I Is Required for Maximum Sensitivity to Camptothecin in a DNA Relaxation Assay. J. Biol. Chem..

[37] Fiorani P., Tesauro C., Mancini G., Chillemi G., D’Annessa I., Graziani G., Tentori L., Muzi A., Desideri A. (2009). Evidence of the crucial role of the linker domain on the catalytic activity of human topoisomerase I by experimental and simulative characterization of the Lys681Ala mutant. Nucleic Acids Res..

[38] Fiorani P., Bruselles A., Falconi M., Chillemi G., Desideri A., Benedetti P. (2003). Single mutation in the linker domain confers protein flexibility and camptothecin resistance to human topoisomerase I. J. Biol. Chem..

[39] D’Annessa I., Tesauro C., Fiorani P., Chillemi G., Castelli S., Vassallo O., Capranico G., Desideri A. (2012). Role of Flexibility in Protein-DNA-Drug Recognition: The Case of Asp677Gly-Val703Ile Topoisomerase Mutant Hypersensitive to Camptothecin. J. Amino Acids.

[40] Tesauro C., Morozzo della Rocca B., Ottaviani A., Coletta A., Zuccaro L., Arnò B., D’Annessa I., Fiorani P., Desideri A. (2013). Molecular mechanism of the camptothecin resistance of Glu710Gly topoisomerase IB mutant analyzed in vitro and in silico. Mol. Cancer.

[41] Cheng C., Kussie P., Pavletich N., Shuman S. (1998). Conservation of structure and mechanism between eukaryotic topoisomerase I and site-specific recombinases. Cell.

[42] Krogh B.O., Shuman S. (2000). Catalytic mechanism of DNA topoisomerase IB. Mol. Cell.

[43] Wang Z., D’Annessa I., Tesauro C., Croce S., Ottaviani A., Fiorani P., Desideri A. (2015). Mutation of Gly717Phe in human topoisomerase 1B has an effect on enzymatic function, reactivity to the camptothecin anticancer drug and on the linker domain orientation. Biochim. Biophys. Acta.

[44] Pommier Y. (2006). Topoisomerase I inhibitors: Camptothecins and beyond. Nat. Rev. Cancer.

[45] Pommier Y. (2013). Drugging Topoisomerases: Lessons and Challenges. ACS Chem. Biol..

[46] Cinelli M.A. (2019). Topoisomerase 1B poisons: Over a half-century of drug leads, clinical candidates, and serendipitous discoveries. Med. Res. Rev..

[47] Strumberg D., Pilon A.A., Smith M., Hickey R., Malkas L., Pommier Y. (2000). Conversion of topoisomerase I cleavage complexes on the leading strand of ribosomal DNA into 5’-phosphorylated DNA double-strand breaks by replication runoff. Mol. Cell. Biol..

[48] Eng W.K., Faucette L., Johnson R.K., Sternglanz R. (1988). Evidence that DNA topoisomerase I is necessary for the cytotoxic effects of camptothecin. Mol. Pharmacol..

[49] Hsiang Y.H., Hertzberg R., Hecht S., Liu L.F. (1985). Camptothecin induces protein-linked DNA breaks via mammalian DNA topoisomerase I. J. Biol. Chem..

[50] Castelli S., Coletta A., D’Annessa I., Fiorani P., Tesauro C., Desideri A. (2012). Interaction between natural compounds and human topoisomerase I. Biol. Chem..

[51] Xin L.-T., Liu L., Shao C.-L., Yu R.-L., Chen F.-L., Yue S.-J., Wang M., Guo Z.-L., Fan Y.-C., Guan H.-S. (2017). Discovery of DNA Topoisomerase I Inhibitors with Low-Cytotoxicity Based on Virtual Screening from Natural Products. Mar. Drugs.

[52] Tillhon M., Guamán Ortiz L.M., Lombardi P., Scovassi A.I. (2012). Berberine: New perspectives for old remedies. Biochem. Pharmacol..

[53] Jadaun A., Subbarao N., Dixit A. (2017). Allosteric inhibition of topoisomerase I by pinostrobin: Molecular docking, spectroscopic and topoisomerase I activity studies. J. Photochem. Photobiol. B Biol..

[54] Honkanen E., Virtanen A.I., Tweit R.C., Dodson R.M. (1960). The Synthesis of Precursor II of Benzoxazolinone Formed in Rye Plants, and the Enzymic Hydrolysis of Precursor I, the Glucoside. Acta Chem. Scand..

[55] Foto E., Özen Ç., Zilifdar F., Tekiner-Gülbaş B., Yıldız İ., Akı-Yalçın E., Diril N., Yalçın İ. (2020). Benzoxazines as new human topoisomerase I inhibitors and potential poisons. DARU J. Pharm. Sci..

[56] Chan A., Chang W.-S., Chen L.-M., Lee C.-M., Chen C.-E., Lin C.-M., Hwang J.-L. (2009). Evodiamine Stabilizes Topoisomerase I-DNA Cleavable Complex to Inhibit Topoisomerase I Activity. Molecules.

[57] El-Atawy M.A., Omar A.Z., Hagar M., Shashira E.M. (2019). Transalkylidation reaction: Green, catalyst-free synthesis of thiosemicarbazones and solving the NMR conflict between their acyclic structure and intramolecular cycloaddition products. Green Chem. Lett. Rev..

[58] Abu N., Ho W.Y., Yeap S.K., Akhtar M.N., Abdullah M.P., Omar A.R., Alitheen N.B. (2013). The flavokawains: Uprising medicinal chalcones. Cancer Cell Int..

[59] Nagle D.G., Ferreira D., Zhou Y.D. (2006). Epigallocatechin-3-gallate (EGCG): Chemical and biomedical perspectives. Phytochemistry.

[60] Berger S.J., Gupta S., Belfi C.A., Gosky D.M., Mukhtar H. (2001). Green tea constituent (-)-epigallocatechin-3-gallate inhibits topoisomerase I activity in human colon carcinoma cells. Biochem. Biophys. Res. Commun..

[61] Singh R., Ahmed S., Islam N., Goldberg V.M., Haqqi T.M. (2002). Epigallocatechin-3-gallate inhibits interleukin-1β-induced expression of nitric oxide synthase and production of nitric oxide in human chondrocytes: Suppression of nuclear factor κB activation by degradation of the inhibitor of nuclear factor κB. Arthritis Rheum..

[62] Kondo T., Ohta T., Igura K., Hara Y., Kaji K. (2002). Tea catechins inhibit angiogenesis in vitro, measured by human endothelial cell growth, migration and tube formation, through inhibition of VEGF receptor binding. Cancer Lett..

[63] Lee J.Y., Moon S.S., Hwang B.K. (2005). Isolation and antifungal activity of kakuol, a propiophenone derivative fromAsarum sieboldii rhizome. Pest Manag. Sci..

[64] Castelli S., Vieira S., D’Annessa I., Katkar P., Musso L., Dallavalle S., Desideri A. (2013). A derivative of the natural compound kakuol affects DNA relaxation of topoisomerase IB inhibiting the cleavage reaction. Arch. Biochem. Biophys..

[65] Neag M.A., Mocan A., Echeverría J., Pop R.M., Bocsan C.I., Crisan G., Buzoianu A.D. (2018). Berberine: Botanical Occurrence, traditional uses, extraction methods, and relevance in cardiovascular, metabolic, hepatic, and renal disorders. Front. Pharmacol..

[66] Xie Q., Johnson B.R., Wenckus C.S., Fayad M.I., Wu C.D. (2012). Efficacy of berberine, an antimicrobial plant alkaloid, as an endodontic irrigant against a mixed-culture biofilm in an in vitro tooth model. J. Endod..

[67] Lou T., Zhang Z., Xi Z., Liu K., Li L., Liu B., Huang F. (2011). Berberine inhibits inflammatory response and ameliorates insulin resistance in hepatocytes. Inflammation.

[68] Lau C.W., Yao X.Q., Chen Z.Y., Ko W.H., Huang Y. (2001). Cardiovascular actions of berberine. Cardiovasc. Drug Rev..

[69] Vieira S., Castelli S., Falconi M., Takarada J., Fiorillo G., Buzzetti F., Lombardi P., Desideri A. (2015). Role of 13-(di)phenylalkyl berberine derivatives in the modulation of the activity of human topoisomerase IB. Int. J. Biol. Macromol..

[70] Christena L.R., Subramaniam S., Vidhyalakshmi M., Mahadevan V., Sivasubramanian A., Nagarajan S. (2015). Dual role of pinostrobin-a flavonoid nutraceutical as an efflux pump inhibitor and antibiofilm agent to mitigate food borne pathogens. RSC Adv..

[71] Patel N.K., Bhutani K.K. (2014). Pinostrobin and Cajanus lactone isolated from *Cajanus cajan* (L.) leaves inhibits TNF-α and IL-1β production: In vitro and in vivo experimentation. Phytomedicine.

[72] Lopez-Martinez L.X., Parkin K.L., Garcia H.S. (2014). Antioxidant and quinone reductase inducing activities of ethanolic fractions from purple maize. LWT Food Sci. Technol..

[73] Smolarz H.D., Bogucka-Kocka A., Mendyk E., Kocki J. (2006). Pinostrobin—An Anti-Leukemic Flavonoid from *Polygonum lapathifolium* L. ssp. nodosum (Pers.) Dans. Zeitschrift fur Naturforsch. Sect. C J. Biosci..

[74] Benson A.A., Daniel H., Wiser R. (1959). A sulfolipid in plants. Proc. Natl. Acad. Sci. USA.

[75] Bharitkar Y.P., Bathini S., Ojha D., Ghosh S., Mukherjee H., Kuotsu K., Chattopadhyay D., Mondal N.B. (2014). Antibacterial and antiviral evaluation of sulfonoquinovosyldiacylglyceride: A glycolipid isolated from *Azadirachta indica* leaves. Lett. Appl. Microbiol..

[76] Jain C.K., Pradhan B.S., Banerjee S., Bikash Mondal N., Majumder S.S., Bhattacharyya M., Chakrabarti S., Roychoudhury S., Kumar Majumder H. (2015). Sulfonoquinovosyl diacylglyceride selectively targets acute lymphoblastic leukemia cells and exerts potent anti-leukemic effects in vivo OPEN. Nat. Publ. Gr..

[77] Tipton C.L., Ming-Chung W., Tsao F.H.C., Chang-chu L.T., Husted R.R. (1973). Biosynthesis of 1,4-benzoxazin-3-ones in Zea mays. Phytochemistry.

[78] Willmott C.J.R., Maxwell A. (1993). A single point mutation in the DNA gyrase A protein greatly reduces binding of fluoroquinolones to the gyrase-DNA complex. Antimicrob. Agents Chemother..

[79] Wu J.-H., Chang F.-R., Hayashi K., Shiraki H., Liaw C.-C., Nakanishi Y., Bastow K.F., Yu D., Chen I.-S., Lee K.-H. (2003). Antitumor agents. Part 218: Cappamensin A, a new In vitro anticancer principle, from *Capparis sikkimensis*. Bioorg. Med. Chem. Lett..

[80] Liao J.F., Chiou W.F., Shen Y.C., Wang G.J., Chen C.F. (2011). Anti-inflammatory and anti-infectious effects of *Evodia rutaecarpa* (Wuzhuyu) and its major bioactive components. Chin. Med..

[81] Jain C., Majumder H., Roychoudhury S. (2016). Natural Compounds as Anticancer Agents Targeting DNA Topoisomerases. Curr. Genom..

[82] Jiang J., Hu C. (2009). Evodiamine: A Novel Anti-Cancer Alkaloid from *Evodia rutaecarpa*. Molecules.

[83] Dong G., Sheng C., Wang S., Miao Z., Yao J., Zhang W. (2010). Selection of evodiamine as a novel topoisomerase i inhibitor by structure-based virtual screening and hit optimization of evodiamine derivatives as antitumor agents. J. Med. Chem..

[84] Spence J.T.J., George J.H. (2011). Structural reassignment of cytosporolides A-C via Biomimetic synthetic studies and reinterpretation of NMR Data. Org. Lett..

[85] Li Y., Niu S., Sun B., Liu S., Liu X., Che Y. (2010). Cytosporolides A-C, antimicrobial meroterpenoids with a unique peroxylactone skeleton from cytospora sp.. Org. Lett..

[86] Otake K., Yamada K., Miura K., Sasazawa Y., Miyazaki S., Niwa Y., Ogura A., Takao K.-I., Simizu S. (2019). Identification of topoisomerases as molecular targets of cytosporolide C and its analog. Bioorganic Med. Chem..

[87] Uivarosi V., Munteanu A. (2017). Flavonoid Complexes as Promising Anticancer Metallodrugs. Flavonoids—From Biosynthesis to Human Health.

[88] Vutey V., Castelli S., D’Annessa I., Sâmia L.B.P., Souza-Fagundes E.M., Beraldo H., Desideri A. (2016). Human topoisomerase IB is a target of a thiosemicarbazone copper(II) complex. Arch. Biochem. Biophys..

[89] Li C.W., Xia M.W., Cui C.B., Peng J.X., Li D.H. (2016). A novel oxaphenalenone, penicimutalidine: Activated production of oxaphenalenones by the diethyl sulphate mutagenesis of marine-derived fungus *Penicillium purpurogenum* G59. RSC Adv..

[90] Andersen R.J., Faulkner D.J., Cun-heng H., Van Duyne G.D., Clardy J. (1985). Metabolites of the Marine *Prosobranch mollusc* lamellaria sp.. J. Am. Chem. Soc..

[91] Tan K.W., Seng H.L., Lim F.S., Cheah S.C., Ng C.H., Koo K.S., Mustafa M.R., Ng S.W., Maah M.J. (2012). Towards a selective cytotoxic agent for prostate cancer: Interaction of zinc complexes of polyhydroxybenzaldehyde thiosemicarbazones with topoisomerase i. Polyhedron.

[92] Rozmer Z., Perjési P. (2016). Naturally occurring chalcones and their biological activities. Phytochem. Rev..

[93] Isa N.M., Abdelwahab S.I., Mohan S., Abdul A.B., Sukari M.A., Taha M.M.E., Syam S., Narrima P., Cheah S.C., Ahmad S. (2012). In vitro anti-inflammatory, cytotoxic and antioxidant activities of boesenbergin A, a chalcone isolated from *Boesenbergia rotunda* (L.) (fingerroot). Brazilian J. Med. Biol. Res..

[94] Ajiboye T.O., Yakubu M.T., Oladiji A.T. (2014). Cytotoxic, Antimutagenic, and Antioxidant Activities of Methanolic Extract and Chalcone Dimers (Lophirones B and C) Derived from *Lophira alata* (Van Tiegh. Ex Keay) Stem Bark. J. Evid. Based Complement. Altern. Med..

[95] Lee D.Y.W., Liu Y. (2003). Molecular structure and stereochemistry of silybin A, silybin B, isosilybin A, and isosilybin B, isolated from *Silybum marianum* (milk thistle). J. Nat. Prod..

[96] Cufí S., Bonavia R., Vazquez-Martin A., Corominas-Faja B., Oliveras-Ferraros C., Cuyàs E., Martin-Castillo B., Barrajón-Catalán E., Visa J., Segura-Carretero A. (2013). Silibinin meglumine, a water-soluble form of milk thistle silymarin, is an orally active anti-cancer agent that impedes the epithelial-to-mesenchymal transition (EMT) in EGFR-mutant non-small-cell lung carcinoma cells. Food Chem. Toxicol..

[97] Kasala E.R., Bodduluru L.N., Madana R.M., Athira K.V., Gogoi R., Barua C.C. (2015). Chemopreventive and therapeutic potential of chrysin in cancer: Mechanistic perspectives. Toxicol. Lett..

[98] León I.E., Cadavid-Vargas J.F., Tiscornia I., Porro V., Castelli S., Katkar P., Desideri A., Bollati-Fogolin M., Etcheverry S.B. (2015). Oxidovanadium(IV) complexes with chrysin and silibinin: Anticancer activity and mechanisms of action in a human colon adenocarcinoma model. J. Biol. Inorg. Chem..

[99] Marco E., Laine W., Tardy C., Lansiaux A., Iwao M., Ishibashi F., Bailly C., Gago F. (2005). Molecular determinants of topoisomerase I poisoning by lamellarins: Comparison with camptothecin and structure-activity relationships. J. Med. Chem..

[100] Perry N.B., Ettouati L., Litaudon M., Blunt J.W., Munro M.H.G., Parkin S., Hope H. (1994). Alkaloids from the antarctic sponge Kirkpatrickia varialosa. Part 1: Variolin b, a new antitumour and antiviral compound. Tetrahedron.

[101] Furrow F.B., Amsler C.D., McClintock J.B., Baker B.J. (2003). Surface sequestration of chemical feeding deterrents in the Antarctic sponge *Latrunculia apicalis* as an optimal defense against sea star spongivory. Mar. Biol..

[102] Beretta G.L., Gatti L., Perego P., Zaffaroni N. (2013). Camptothecin resistance in cancer: Insights into the molecular mechanisms of a DNA-damaging drug. Curr. Med. Chem..

[103] Moon J.Y., Song I.C., Ko Y.B., Lee H.J. (2018). The combination of cisplatin and topotecan as a second-line treatment for patients with advanced/recurrent uterine cervix cancer. Medicine.

[104] Fang S.-M., Wu C.-J., Li C.-W., Cui C.-B. (2014). A Practical Strategy to Discover New Antitumor Compounds by Activating Silent Metabolite Production in Fungi by Diethyl Sulphate Mutagenesis. Mar. Drugs.

[105] Djerassi C., Lam W.K. (1991). Sponge Phospholipids. Acc. Chem. Res..

[106] Carballeira N.M., Montano N., Amador L.A., Rodríguez A.D., Golovko M.Y., Golovko S.A., Reguera R.M., Álvarez-Velilla R., Balaña-Fouce R. (2016). Novel Very Long-Chain α-Methoxylated Δ5,9 Fatty Acids from the Sponge Asteropus Niger Are Effective Inhibitors of Topoisomerases IB. Lipids.

[107] Quesada A.R., García Grávalos M.D., Fernández Puentes J.L. (1996). Polyaromatic alkaloids from marine invertebrates as cytotoxic compounds and inhibitors of multidrug resistance caused by P-glycoprotein. Br. J. Cancer.

[108] Imperatore C., Aiello A., D’Aniello F., Senese M., Menna M. (2014). Alkaloids from marine invertebrates as important leads for anticancer drugs discovery and development. Molecules.

[109] Facompré M., Tardy C., Bal-Mahieu C., Colson P., Perez C., Manzanares I., Cuevas C., Bailly C. (2003). Lamellarin D: A Novel Potent Inhibitor of Topoisomerase I. Cancer Res..

[110] Liu M., Wang G., Xiao L., Xu A., Liu X., Xu P., Lin X. (2014). Bis(2,3-dibromo-4,5-dihydroxybenzyl) ether, a marine algae derived bromophenol, inhibits the growth of Botrytis cinerea and interacts with DNA molecules. Mar. Drugs.

[111] Xu N., Fan X., Yan X., Li X., Niu R., Tseng C.K. (2003). Antibacterial bromophenols from the marine red alga Rhodomela confervoides. Phytochemistry.

[112] Shi D., Xu F., He J., Li J., Fan X., Han L. (2008). Inhibition of bromophenols against PTP1B and anti-hyperglycemic effect of Rhodomela confervoides extract in diabetic rats. Chin. Sci. Bull..

[113] Liu M., Zhang W., Wei J., Qiu L., Lin X. (2012). Marine bromophenol bis(2,3-dibromo-4,5-dihydroxybenzyl) ether, induces mitochondrial apoptosis in K562 cells and inhibits topoisomerase I in vitro. Toxicol. Lett..

[114] Canals A., Arribas-Bosacoma R., Albericio F., Álvarez M., Aymamí J., Coll M. (2017). Intercalative DNA binding of the marine anticancer drug variolin B. Sci. Rep..

[115] Simone M., Erba E., Damia G., Vikhanskaya F., Di Francesco A.M., Riccardi R., Bailly C., Cuevas C., Fernandez Sousa-Faro J.M., D’Incalci M. (2005). Variolin B and its derivate deoxy-variolin B: New marine natural compounds with cyclin-dependent kinase inhibitor activity. Eur. J. Cancer.

[116] Alvarez B., Bergquist P.R., Battershill C.N. (2002). Taxonomic revision of the genus Latrunculia Du Bocage (Porifera: Demospongiae: Latrunculiidae) in New Zealand. N. Z. J. Mar. Freshw. Res..

[117] Reyes F., Martín R., Rueda A., Fernández R., Montalvo D., Gómez C., Sánchez-Puelles J.M. (2004). Discorhabdins I and L, Cytotoxic Alkaloids from the Sponge *Latrunculia brevis*. J. Nat. Prod..

[118] Li F., Peifer C., Janussen D., Tasdemir D. (2019). New discorhabdin alkaloids from the antarctic deep-sea sponge latrunculia biformis. Mar. Drugs.

[119] Kawato Y., Aonuma M., Hirota Y., Kuga H., Sato K. (1991). Intracellular Roles of SN-38, a Metabolite of the Camptothecin Derivative CPT-11, in the Antitumor Effect of CPT-11. Cancer Res..

[120] Crea F., Giovannetti E., Cortesi F., Mey V., Nannizzi S., Gallegos Ruiz M.I., Ricciardi S., Del Tacca M., Peters G.J., Danesi R. (2009). Epigenetic mechanisms of irinotecan sensitivity in colorectal cancer cell lines. Mol. Cancer Ther..

[121] Kingsbury W.D., Boehm J.C., Jakas D.R., Holden K.G., Gallagher G., Caranfa M.J., McCabe F.L., Faucette L.F., Johnson R.K., Hertzberg R.P. (1991). Synthesis of Water-Soluble (Aminoalkyl)camptothecin Analogues: Inhibition of Topoisomerase I and Antitumor Activity. J. Med. Chem..

[122] Paton F., Paulden M., Saramago P., Manca A., Misso K., Palmer S., Eastwood A. (2010). Topotecan for the treatment of recurrent and stage IVB carcinoma of the cervix. Health Technol. Assess..

[123] Lee D.H., Kim S.W., Suh C., Lee J.S., Lee J.H., Lee S.J., Ryoo B.Y., Park K., Kim J.S., Heo D.S. (2008). Belotecan, new camptothecin analogue, is active in patients with small-cell lung cancer: Results of a multicenter early phase II study. Ann. Oncol..

[124] Crul M. (2003). CKD-602 Chong Kun Dang. Curr. Opin. Investig. Drugs.

[125] Kim G.M., Kim Y.S., Ae Kang Y., Jeong J.H., Kim S.M., Hong Y.K., Sung J.H., Lim S.T., Kim J.H., Kim S.K. (2012). Efficacy and toxicity of belotecan for relapsed or refractory small cell lung cancer patients. J. Thorac. Oncol..

[126] Hun Choi C., Lee Y.-Y., Song T.-J., Park H.-S., Kyu Kim M., Kim T.-J., Lee J.-W., Lee J.-H., Bae D.-S., Kim B.-G. (2011). Phase II Study of Belotecan, a Camptothecin Analogue, in Combination with Carboplatin for the Treatment of Recurrent Ovarian Cancer. Cancer.

[127] Rasheed Z.A., Rubin E.H. (2003). Mechanisms of resistance to topoisomerase I-targeting drugs. Oncogene.

[128] Li F., Jiang T., Li Q., Ling X. (2017). Camptothecin (CPT) and its derivatives are known to target topoisomerase I (Top1) as their mechanism of action: Did we miss something in CPT analogue molecular targets for treating human disease such as cancer?. Am. J. Cancer Res..

[129] Topoisomerase I Inhibitor—List Results—ClinicalTrials.gov. https://clinicaltrials.gov/ct2/results?cond=&term=topoisomerase+I+inhibitor&cntry=&state=&city=&dist=&Search=Search.

[130] Cao Z., Kozielski A., Liu X., Wang Y., Vardeman D., Giovanella B. (2009). Crystalline camptothecin-20(S)-O-propionate hydrate: A novel anticancer agent with strong activity against 19 human tumor xenografts. Cancer Res..

[131] Beck D.E., Abdelmalak M., Lv W., Reddy P.V.N., Tender G.S., O’Neill E., Agama K., Marchand C., Pommier Y., Cushman M. (2015). Discovery of potent indenoisoquinoline topoisomerase i poisons lacking the 3-nitro toxicophore. J. Med. Chem..

[132] Yu Q., Chen Y., Yang H., Zhang H.L., Agama K., Pommier Y., An L.K. (2019). The antitumor activity of CYB-L10, a human topoisomerase IB catalytic inhibitor. J. Enzyme Inhib. Med. Chem..

[133] Arakawa Y. (2015). DNA topoisomerase I-targeting drugs. Nihon Rinsho..

[134] Ireton G.C. (1996). The Domain Organization of Human Topoisomerase I. J. Biol. Chem..

[135] Beutler J.A. (2009). Natural products as a foundation for drug discovery. Curr. Protoc. Pharmacol..

